# Integrating neuroscience and learning: now’s the time...

**DOI:** 10.1038/npjscilearn.2016.7

**Published:** 2016-05-11

**Authors:** Pankaj Sah, Michael Fanselow, John Hattie, Susan Magsamen, Jason Mattingley, Gregory Quirk, Stephen Williams

**Affiliations:** 1 Queensland Brain Institute, The University of Queensland, Brisbane, QLN, Australia; 2 University of California, Los Angeles, CA, USA; 3 University of Melbourne, Melbourne, VIC, Australia; 4 The Brain Science Institute and The Science of Learning Institute, Johns Hopkins University, Baltimore, MD, USA; 5 Houghton Mifflin Harcourt, Boston, MA, USA; 6 School of Psychology, The University of Queensland, Brisbane, QLN, Australia; 7 University of Puerto Rico, San Juan, Puerto Rico

The ability to learn and to retrieve information from memory arose early in the evolution of animals, and is present across all species, from humans to the simple roundworm (*C. elegans*). The capacity to learn is critical for survival, whether it be to find food and avoid predators, or to engage in effective social interactions and be productive in the workplace. Our ability to increase human potential is linked to our ability to learn at all life stages. Using experimental models, in recent decades, the fields of neuroscience and experimental psychology have made great strides in understanding how learning occurs, both in terms of cognitive processes, and their underlying neural mechanisms. These studies are providing insight into questions about learning, and possible translational solutions from the cradle to the classroom. For example, this work is beginning to provide an understanding of disorders in memory formation, storage and retrieval, such as with ageing and dementia^1^. It is also exploring stress, sleep and fear as factors that diminish learning.

While it is widely believed that the same mechanisms and systems underpin learning at large—in the classroom and in informal learning environments—minimal progress has been made to advance the translation and practice of this information. In other words, the results of experimental studies are inconsistently and often inaccurately informing educational practice. Further, educational practice is not informing basic research in meaningful ways—an essential two way street.

Traditional learning environments are largely driven by pedagogy, with its roots in social and developmental theory with little or no input from neuroscience or psychology. Likewise, most laboratory experiments designed to investigate the neural and psychological processes that regulate learning have not drawn upon the wealth of knowledge accumulated by teachers and educators in real-world learning contexts. One reason for these failures of translation is that neuroscientists, psychologists and educators speak different langauges and have different approaches when it comes to thinking about learning. Another is that each discipline tends to focus on its own unique level of explanation for various learning effects, and these can be hard to relate to one another. For example, when a teacher or parent notices an anxious child struggling to learn a new mathematical concept, how is the neuroscientist to design an experiment to test this at the level of neurons, systems and synapses? Likewise, can the teacher harness the latest advances in understanding the nature of synaptic mechanisms of learning to help her students solve fractions? Would this even matter? Can an educator or parent begin to have a new perspective on how the brain learns to help inform pedagogy and skill development? Can researchers work together with educators to create meaningful translations of research that increase every human’s learning potential?

Our goal for *npj Science of Learning* is to overcome some of these barriers and create common ground by providing an open-access forum for all people interested in learning to begin to talk the same language and to share their ideas from across the spectrum of relevant disciplines. As editors, our goal is to provide a forum for discussion of advances at all levels that contribute to understanding learning. We aim to publish cutting edge research on the mechanisms that underpin and influence learning and memory formation in experimental systems, as well as the pedagogical and social factors that influence education. The open-access nature of this journal facilitates the support of scientists, educators, informal learning advocates and policy makers to drive experimental investigations, and guide the practice and assessment of education. We aim to publish findings in the functional, cellular, molecular, cognitive and systems studies of learning and memory formation, as well as the ideas and thinking of education theory. It is the link that matters for *npj Science of Learning*. To support cross-talk between disciplines, a lay summary will accompany each research article, to make the findings more accessible to both scientists, formal educators, advocates, informal educators and policy makers.

This diversity is refected in the scope of articles in this first issue. Long-term potentiation (or LTP) is a form of synaptic plasticity that is believed to be the cellular basis for memory storage. As with memory formation and consolidation, LTP has early short and late persistent phases, and different molecular mechanisms mediate the short and persistent forms of LTP. Pang *et al.*^2^ show that just as with the early phase of LTP, the persistent form also has two phases: induction and mainteinance. They show the two forms require differential cleavage of the protein brian-derived neurotropic factor. Patricia Alexander^3^ reviews the current state of relational thinking and reasoning, and suggests ways in which these different techniques could be used in the classroom. Levitan *et al.*^4^ show that protein synthesis plays distinct roles in memory. Using conditioned taste aversion, they show that while formation of long-term memories requires protein synthesis, keeping these representations in long-term memory requires a reduction in protein synthesis.

To be successful, this highly interdisciplinary and diverse community needs to come together to talk about needs and different approaches. We need to create a common language with clarity on goals and outcomes. We encourage you to participate in the creation of this community that has the potential to transfer learning. We invite you to make *npj Science of Learning* your forum of choice.
